# Anti-*Candida albicans* Activity of Ononin and Other Secondary Metabolites from *Platonia Insignis* MART

**DOI:** 10.3390/metabo12111014

**Published:** 2022-10-24

**Authors:** Anderson França da Silva, Josivan Regis Farias, Danielle Cristine Gomes Franco, Andrea Araruna Galiza, Elizangela Pestana Motta, Aluísio da Silva Oliveira, Cleydlenne Costa Vasconcelos, Maria do Socorro de Sousa Cartágenes, Claudia Quintino da Rocha, Mayara Cristina Pinto da Silva, Alberto Jorge Oliveira Lopes, Flavia Raquel Fernandes do Nascimento, Cristina Andrade Monteiro, Rosane Nassar Meireles Guerra

**Affiliations:** 1 Laboratory of Immunophysiolgy, Federal University of Maranhão, São Luís 65080-805, Brazil; 2 Program in Biotechnology-RENORBIO, Federal University of Maranhão, São Luís 65080-805, Brazil; 3 Program in Health Sciences, Federal University of Maranhão, São Luís 65080-805, Brazil; 4 Laboratory of Experimental Study of Pain, Department of Physiological Sciences, Federal University of Maranhão, São Luís 65080-805, Brazil; 5 Department of Chemistry, Federal University of Maranhão, São Luís 65080-805, Brazil; 6 Federal Institute of Science Education and Technology of Maranhão-Campus Santa Inês, Santa Inês 65300-000, Brazil; 7 Department of Biology, Federal Institute of Science Education and Technology of Maranhão, São Luís 65030-005, Brazil

**Keywords:** *Candida albicans*, ononin, molecular docking, *Tenebrio molitor*, *Platonia insignis*, antifungal, medicinal chemistry, new drugs

## Abstract

*Candida albicans* is a human pathogen that is part of the healthy microbiome. However, it is often associated with opportunistic fungal infections. The treatment of these infections is challenging because prolonged exposure to antifungal drugs can culminate in fungal resistance during therapy, and there is a limited number of available drugs. Therefore, this study investigated the antifungal activity of ononin by in silico and in vitro assays, and in *Tenebrio molitor* as an alternative in vivo model of infection caused by *C. albicans*. Ononin is an isoflavone glycoside derived from formononetin that has various biological activities. According in silico evaluation, ononin showed the best electron affinity in molecular docking with CaCYP51, with a binding free energy of −10.89 kcal/mol, superior to that of the antifungal drugs fluconazole and posaconazole. The ononin + CaCYP51 complex formed hydrogen bonds with Tyr132, Ser378, Phe380, and Met508, as well as hydrophobic connections with Tyr118, Leu121, Phe126, Leu131, Ile304, and Leu309, and interactions with the heme group. Ononin exerted anti-*Candida albicans* activity, with MIC between 3.9 and 7.8 µg/mL, and inhibited young and mature biofilms, with a reduction in cell density and metabolic activity of 50 to 80%. The compound was not cytotoxic to sheep red blood cells at concentrations up to 1000 µg/mL. Larvae of the mealworm *T. molitor* were used as an alternative in vivo model of *C. albicans* infection. Ononin was able to prolong larval survival at concentrations of 0.5, 1, and 5 mg/kg, and was not toxic up to a concentration of 20 mg/kg. Moreover, ononin reduced the fungal charge in treated animals. In conclusion, our results suggest that ononin has anti-*Candida albicans* activity and is a potential candidate for the development of new therapeutic alternatives.

## 1. Introduction

*Candida albicans* is part of the healthy human microbiome, and is the fungus most frequently associated with asymptomatic colonization of the human gut, vaginal, and skin microbiomes [1,2]. In most immunocompetent individuals, this fungus lives in harmony with other members of the microbiota [1]. Certain situations, such as pH and nutritional alterations, antibiotic use, and immune suppression (patients receiving chemotherapy or patients with AIDS) can trigger proliferation of the fungus and give rise to infections that can spread through the bloodstream and cause severe infections whose mortality rates are higher than 40% [2,3].

A common treatment for these infections is the administration of a broad-spectrum antifungal agent. Among the antimicrobials destined for this purpose, azoles are by far the most widely used, both in isolation [3,4] or in synergism with other drugs and compounds [5]. These antifungal agents act by inhibiting the cytochrome P450 enzyme lanosterol 14α-demethylase (CYP51), which is encoded by the ERG11 gene and converts lanosterol to ergosterol [3,5,6]. This enzyme contains an iron protoporphyrin complex (heme group) in its active site. This group is dependent on cytochrome P450, a key enzyme in the synthesis of sterols. Azoles bind to the iron of protoporphyrin and block the ergosterol biosynthesis pathway, with the consequent accumulation of 14α-methylated sterols that are toxic and inhibit fungal growth and replication [6].

Studies have shown that prolonged exposure to antifungal agents, especially those of the azole class, can culminate in fungal resistance [6]. In addition, *C. albicans* can form highly structured biofilms that contain yeast-like cells, pseudohyphae, and hyphae embedded in an extracellular matrix. These biofilms are known to develop on abiotic and biotic surfaces, and are difficult to control [2,7]. *Candida* biofilms can colonize implanted medical devices such as catheters, pacemakers, heart valves, and prostheses, and can form on host surfaces such as mucosae, epithelial cell linings, and parenchymatous organs [3,8]. Furthermore, conventional antifungal drugs can cause various adverse effects such as gastrointestinal discomfort, skin rash, and hepatotoxicity [9]. Therefore, new therapeutic approaches for the treatment of *C. albicans* infections are urgently needed.

In a study conducted by our research group [10], the hydroethanolic extract and ethyl acetate fraction of *Platonia* *insignis* Mart. exhibited antifungal activity. *Platonia insignis* (Clusiaceae family) is a species of the genus *Platonia*, and is popularly known as “bacurizeiro” in the Brazilian Amazon region, where it is possible to find high densities of this tree [9]. The main compounds identified by LC-ESI-IT-MS were quinic acid, ononin, orientin, vitexin, and fukugentin. Ononin is an isoflavone glycoside derived from formononetin that has various biological activities. This compound has been identified in several plant species, including the stem of *Millettia nitida* var. hirsutissima [11], rhizomes and roots of *Smilax scobinicaulis* [12], roots of *Astragali radix* [13], and leaves of *Platonia insignis* Mart. [10], and is one of the major isoflavonoids. Different biological activities of ononin have been reported, such as antioxidant, anti-inflammatory, antiviral, and anti-pulmonary fibrosis properties [14,15,16,17,18], as well as in vivo anti-inflammatory activity [19]. Furthermore, ononin exerts antibacterial activity [20,21], including activity against *Escherichia coli* [22]; however, there are no studies describing its antifungal activity.

There are several software that facilitate the analysis of possible drugs, thus they may contribute to identify new therapeutic drugs [6]. Molecular docking (MD) is a method that can predict the orientation of a ligand to its receptor (protein) to form a stable complex. However, we found no MD studies that established the binding mode of ononin in the active site of CYP51, the target of antifungal azoles. Therefore, the hypothesis of this study was that ononin, a substance present in *P. insignis* extract, is associated with the antifungal effect. Based on this, the present study investigated the antifungal activity of ononin by in silico and in vitro assays, and in *Tenebrio molitor* as an alternative in vivo model of infection caused by *Candida albicans.* In addition, we also investigated the biological and toxicological activity of ononin in silico.

## 2. Materials and Methods

### 2.1. Vegetal Material and Extract

The leaves of *Platonia insignis* were collected in the municipality of São Luís, Maranhão, Brazil (April–May 2019) and identified in the Herbarium of Maranhão from the Federal University of Maranhão, São Luís, Brazil (Voucher specimen 9722, SISGen A1ED5A9). The leaves were dried in an oven at 40 °C for 3 days, and then dried at room temperature for another 4 days. After that, the leaves were ground in a mill and their crude extract was extracted by maceration in 70% ethanol for 24 h according to a previously described technique [10].

### 2.2. Compounds

Fluconazole (Sigma-Aldrich, São Paulo, Brazil) was prepared in deionized water. Ononin (CAS number 486-62-4), also purchased from Sigma-Aldrich (São Paulo, Brazil), was prepared in a stock solution of dimethyl sulfoxide (DMSO, Merck, São Paulo, Brazil) at 5 mg/mL. The structure of ononin and the physicochemical properties of ononin and fluconazole are shown in the Supplementary Materials.

### 2.3. In Silico Study

#### 2.3.1. Structure of the Compounds and Receptor

A previous study from our group [10] identified the following compounds by LC-ESI-IT-MS in the hydroethanolic extract and ethyl acetate fraction of *Platonia insignis* Mart.: quinic acid, ononin, orientin, vitexin, and fukugentin. The three-dimensional (3D) structures of these compounds were visualized with GaussView 5.0.8 [23]. The geometric and vibrational properties were calculated (optimized) in a vacuum by applying the density functional theory (DFT) method, using a combination of the B3LYP hybrid functional and 6–31 ++ G (d, *p*) basis sets. Calculations were performed using Gaussian 09 [24].

The 3D structure of *C. albicans* 14α-demethylase (CaCYP51) was obtained from the Protein Data Bank (PDB) (#5FSA), and resolved by X-ray crystallography at a resolution of 2.86 Å. The antifungal agent posaconazole present in the crystal and other molecules were removed, maintaining only one of the two homologous chains together with the heme group.

#### 2.3.2. Molecular Docking

The Autodock 4.2 package [25,26] was used for all docking procedures. The structures of CaCYP51 and of the ligands were prepared for MD calculations using AutoDock Tools (ADT), version 1.5.6 [27]. The structure of CaCYP51 was considered rigid, while that of each ligand was assumed to be flexible. The partial Gasteiger charges were calculated after the addition of all hydrogens. Nonpolar hydrogens of CaCYP51 and of the ligands were then merged. A cubic box with 80 × 80 × 80 points and spacing of 0.35 Å between grid points was generated for the entire target protein. The grid box was centered in the Fe atom of the heme group of CaCPY51. The Lamarckian genetic algorithm (LGA) for global searching and the pseudo-Solis and Wets local searches were used for MD. Each ligand was submitted to 100 independent runs of docking simulations. Standard values were defined for the remaining docking parameters. The initial coordinates of the interactions between CaCYP51 metabolites and the compounds studied were chosen using as criterion the lowest energy conformation combined with visual inspection [28].

#### 2.3.3. Prediction of Biological Activities 

The biological activities of ononin and fluconazole (standard drug) were evaluated using PASS Online (www.way2drug.com/passonline (accessed on 05 May 2022)), considering the biological activity as “active” (Pa) or “inactive” (Pi), based on the estimated probability that varies from zero to one. The results of PASS prediction considered only the biological activities with Pa > Pi, and data were as described previously [29], taking into account the following values:

i. Pa > 0.7—the substance probably exhibits biological activity, and there is also a high probability of this compound showing analogy with a known pharmaceutical.
ii. 0.5< Pa < 0.7—the compound may have biological activity, but the substance differs from known drugs.
iii. Pa < 0.5—the chance of finding biological activity is lower, but the chance of finding a structurally new compound is greater.

#### 2.3.4. Prediction of Pharmacokinetic Characteristics and the Toxic Effects of Ononin

The prediction of the characteristics of gastrointestinal absorption of the compounds, and their permeability through the blood–brain barrier, was performed by SwissADME program [30].

The possible toxic effects of ononin was performed using Osíris (©Idorsia Pharmaceuticals Ltd., Allschwil, Switzerland) (www.organic-chemistry.org/prog/peo/drugScore.html) [31]. 

The toxicity parameters were predicted by comparing the chemical structures of the compounds with a database containing commercially available drugs and commercially available compounds. The toxic effects obtained were classified as mutagenic, tumorigenic, irritating, and effects on the reproductive system [32].

#### 2.3.5. In Silico Analysis of Ononin Toxicity

To assess the hepatic toxicity of ononin, we used SuperCYPsPred (http://insilico-cyp.charite.de/SuperCYPsPred (accessed on 5 May 2022)), which includes machine learning models based on the random forest algorithm and different types of data sampling methods. The models presented in SuperCYPsPred discriminate between inhibitors and noninhibitors for the five main CYP450 isoforms. Fragment-based and structural similarity approaches were used to evaluate the applicability domain of the models, in addition to predicting a specific compound as active (inhibitor) or inactive (non-inhibitor) for a defined CYP isoform [29].

#### 2.3.6. LD_50_ Prediction 

The lethal dose (LD_50_) prediction was performed used using GUSAR software (http://way2drug.com/mg/index.php (accessed on 5 May 2022)) [33]. The GUSAR toxicity values were calculated on the Log10 scale to predict the LD_50_ (mg/kg) after intravenous and oral administration for rats.

The toxicity classes were defined according to the Globally Harmonized System of Classification (GHS) according to the values expressed in mg/kg [20].

Class I: fatal if swallowed (LD_50_ ≤ 5).
Class II: fatal by ingestion (5 < LD_50_ ≤ 50).
Class III: toxic by ingestion (50 < LD_50_ ≤ 300).
Class IV: harmful by ingestion (300 < LD_50_ ≤ 2000).
Class V: may be harmful if ingested (2000 < LD_50_ ≤ 5000).
Class VI: nontoxic (LD_50_ > 5000).

### 2.4. Anti-Candida albicans Activity

Three *C. albicans* strains were used for this study: two vaginal isolates, one resistant to fluconazole (CaR) and one sensitive to the drug (CaS), and a reference strain from the American Type Culture Collection (ATCC 10231). The clinical (vaginal) isolates were obtained after approval of the protocol by the Ethics Committee of the Federal University of Maranhão (UFMA) (Approval No. 3.956.307/2019). The isolates are part of the collection of the Laboratory of Immunophysiology, UFMA, São Luís-MA, Brazil, and are maintained in 50% glycerol at −20 °C. The CaR and CaS isolates were identified by matrix-assisted laser desorption ionization mass spectrometry (MALDI-TOF MS, Microflex, Bruker, São Paulo, Brazil), and their fluconazole profile was obtained by an automated method (VITEK^®^2 Compact, BioMérieux, São Paulo, Brazil) and confirmed by the Kirby–Bauer method according to standard M27-A3 [34].

#### 2.4.1. Minimum Inhibitory Concentration

The minimum inhibitory concentrations (MIC) of ononin and fluconazole were determined using the standardized antifungal microdilution susceptibility test proposed by the Clinical and Laboratory Standards Institute (CLSI, standard M27-A3) [34]. The microorganisms were reactivated on Sabouraud dextrose agar (SDA; Kasvi, Italy) for 24 h at 37 °C. The inoculum was prepared in NaCl (0.85%) from colonies isolated from 18–24-h cultures and adjusted in a Neubauer chamber to a density corresponding to 0.5 on the McFarland scale. For the microdilution tests, the suspension was diluted in RPMI 1640 medium with glutamine and without bicarbonate (Sigma-Aldrich, St. Louis, MO, USA), and buffered with MOPS (morpholinepropanesulfonic acid; Sigma Chemical, St. Louis, MO, USA) at a pH of 7.0 ± 0.2 to obtain a concentration of 1 × 10^3^ to 5 × 10^3^ CFU/mL (CLSI, 2008). The final concentrations ranged from 0.24 to 500 µg/mL for ononin and from 0.125 to 0 128 µg/mL for fluconazole. The MIC was defined as a 50% reduction in visible growth of the microorganism compared to the growth control. DMSO (1%) was used as vehicle control.

#### 2.4.2. Minimum Fungicidal Concentration

The minimum fungicidal concentration (MFC) was defined as the concentration of the antifungal agent that inhibited the growth of colonies. For the determination of the MFC, 10 µL was removed from all wells of the standard MIC plates and transferred to Petri dishes containing SDA. The plates were incubated for 24–48 h at 37 °C before colony counting. All tests were carried out in triplicate and repeated three times.

#### 2.4.3. Cell Growth Kinetics

This assay was carried according to [35], with modifications. Cultures of the *C. albicans* strains (ATCC 10231, CaR and CaS) grown for 18–24 h on SDA were used. An initial inoculum of 5 × 10^3^ CFU/mL was prepared in fresh RPMI, and ononin was added at concentrations of ¼ MIC, ½ MIC, and MIC. The cultures were incubated at 37 °C. Aliquots were removed after 0, 3, 6, 9, 12, 24, 36, and 48 h. Growth was monitored by measuring the optical density at 600 nm (Softmax^®®^ Pro), plotted against time in hours. DMSO (1%) was used as vehicle control. The assays were performed in six replicates on three different occasions.

#### 2.4.4. Antibiofilm Activity

The in vitro biofilm formation assay was carried out as described previously [4,36,37] with some modifications. Briefly, *C. albicans* ATCC 10231 and CaR cells were incubated on SDA for 18–24 h at 37 °C. The cells were then collected by centrifugation at 3000 g and washed twice in sterile phosphate-buffered saline (PBS). The pellets were suspended in MOPS-buffered RPMI 1640 medium. The inoculum was adjusted to an optical density of 0.5 (~1 × 10^7^ cells/mL) at 600 nm in 200 µL, added to a 96-well flat-bottom plate, and left to stand for 90 min at 37 °C for adhesion. After this period, the supernatant was aspirated carefully to remove nonadherent cells and washed twice with 200 µL PBS. Next, 200 µL of fresh medium with or without the antifungal agent was added to the wells. The following antifungal concentrations were used in this assay: MIC, 2 × MIC, 4 × MIC, and 8 × MIC. After the addition of the antifungal agents, the plates were incubated for 24 or 48 h at 37 °C. For the evaluation of young biofilms, the plates were incubated in the presence of the antifungals for 24 h. Mature biofilms were left to grow for 24 h at 37 °C in the absence of antifungal agents. Next, the old medium was replaced with fresh medium with or without antifungals and incubated for an additional 24 h at 37 °C. After this period, the supernatant was removed and the plates were read at an optical density of 600 nm [36,38,39].

Metabolic activity was analyzed by a colorimetric assay that measures the reduction of 3-methyl-(4–5-dimethylthiazol-2-yl)-2,5-diphenyltetrazolium salt (MTT; Sigma-Aldrich, St. Louis, MO, USA) to formazan according to [10]. After incubation for 24 or 48 h, the biofilms were washed twice with PBS, 100 µL MTT (5 mg/mL) was added, and the plates were incubated for 4 h protected from light. The supernatants were removed, 100 µL DMSO was added, and the plates were incubated for 10 min protected from light. The plates were read in a microplate reader (Softmax^®®^ Pro) at an optical density of 540 nm. The assays were performed in eight replicates on three different occasions.

### 2.5. Hemolytic Activity

Defibrinated sheep blood (EB FARMA, Rio de Janeiro, Brazil) was used for this assay. Red blood cells were isolated by centrifugation at 290× *g* for 10 min at 4 °C. The cells were washed three times with PBS (pH 7.4) and resuspended in 2% PBS (*v*/*v*). Next, 100 µL of the red blood cell suspension containing 3.9 to 1000 µg/mL of ononin was added to 96-well flat-bottom microplates. Total hemolysis was achieved with concentrations of Triton X-100 of 0.1 to 1% (Sigma-Aldrich, St. Louis, MO, USA), while PBS was used as a negative control. DMSO (1%) was used as a vehicle control. After incubation for 60 min at room temperature, the cells were centrifuged at 300× *g* for 10 min at 4 °C, and the supernatant was read in a microplate reader (Softmax^®®^ Pro) at 540 nm [10]. Relative hemolytic activity was calculated in relation to the Triton X-100 control using the following formula:

Relative hemolytic activity (“%”) = ((As − Ab))/((Ac − Ab)) × 100(1)

where Ab is the absorbance of the control (blank, without ononin), As is the absorbance in the presence of ononin, and Ac is the absorbance in the presence of Triton X-100. The assays were carried out three times on different occasions, with six replicates per condition.

### 2.6. In Vivo Assay Using Tenebrio molitor

Early-stage *Tenebrio molitor* larvae (~200 mg) selected based on similarity in size and exhibiting no apparent color alterations were used in all experiments [38,39]. The larvae were placed in sterile Petri dishes for 24 h before the experiments for acclimatization and incubated at 37 °C protected from light. Larvae with dark spots or apparent myelinization were excluded.

#### 2.6.1. Toxicity of Ononin in *Tenebrio molitor*

Ononin at concentrations of 0.1, 0.5, 1, 5, 10, and 20 mg/kg was injected directly into the hemocoel of *T. molitor* (*n* = 20 larvae) with a Hamilton syringe (Hamilton, FL, USA), in the third or fourth sternite of the ventral abdomen previously cleaned with 70% alcohol. A group receiving PBS was included as negative control. The larvae were incubated at 37 °C and mortality was evaluated every 24 h for 7 days. Death was defined as the complete loss of movement and absence of response to physical stimuli using tweezers. The experiments were repeated three times on different occasions.

#### 2.6.2. Survival Assay

*Candida albicans* (ATCC 10231) yeasts were cultured for 18 to 24 h on SDA and then suspended in sterile PBS. Inocula were standardized in a Neubauer counting chamber and concentrations of 5 × 10^4^, 1 × 10^5^, 2.5 × 10^5^, 5 × 10^5^, and 1 × 10^6^ cells/larva (5 µL) were administered with a Hamilton syringe (Hamilton, USA) directly into the hemocoel, in the third or fourth sternite of the ventral abdomen previously cleaned with 70% alcohol. The infected larvae were incubated at 37 °C and monitored for 7 days. Larval death was monitored every 24 h by visual inspection of their color (presence of spots on the body) and absence of movement when touched with tweezers. Groups of 20 larvae and a control group receiving PBS were used for each condition of the study. The assays were repeated at least three times.

#### 2.6.3. Efficacy of Ononin in the Survival of *Tenebrio molitor* Infected with *Candida albicans*

The larvae were randomly divided into groups (*n* = 20 larvae) on sterile Petri dishes and kept at 37 °C in the dark throughout the experiment. An inoculum (5 µL) of 5 × 10^5^ cells/larva of *C. albicans* (ATCC 10231) was injected into the larvae. After 3 h of incubation at 37 °C, the larvae received a single dose of 5 µL ononin (0.5, 1, and 5 mg/kg) and the plates were again incubated at 37 °C. Infected larvae inoculated with vehicle (PBS) were used as a positive control, while uninfected larvae inoculated with PBS served as a negative control. The mortality rates of each group were determined every 24 h for 7 days, evaluating color changes and the absence of movement when touched with tweezers. The assays were performed in three replicates on different occasions.

#### 2.6.4. Determination of Fungal Load

The colony counting method was used to assess the effect of ononin on the fungal load of larvae infected with *C. albicans* (ATCC 10231). The animals were randomly divided into five groups (*n* = 15 larvae) and a previously standardized sublethal inoculum of 5 × 10^4^ cells/larva was used for infection, as described in the previous item. Five larvae of each group were removed randomly every 24 h for 3 days, washed with 70% alcohol, sectioned with a scalpel, triturated, and homogenized in 10 mL of a sterile PBS–chloramphenicol solution. Serial tenfold dilutions were prepared from the homogenate of each group and 10 µL of each dilution was inoculated into SDA–chloramphenicol. The plates were incubated for 24–48 h at 37 °C and the colonies were then enumerated. Each dilution was inoculated using five replicates and the assays were performed on three different occasions.

### 2.7. Statistical Analysis

GraphPad Prism 9.0 software (La Jolla CA, USA) was used for graphical and statistical analyses. The results are expressed as mean ± standard deviation. Statistical analysis was performed using Student’s t-test. The survival curves were analyzed by the log-rank test. A *p* value < 0.05 was considered statistically significant.

## 3. Results

### 3.1. Ononin Exhibits Affinity for Candida albicans CaYP51

Quinic acid, ononin, orientin, vitexin, and fukugentin identified by LC-ESI-IT-MS in the hydroethanolic extract and ethyl acetate fraction of *P. insignis* were used for MD. Ononin exhibited an affinity for CaCYP51 in MD, with a binding free energy of −10.89 kcal/mol and an inhibition constant of 0.01 μM. In addition to the compounds present in the extract, MD analysis of the commercial antifungal agents posaconazole and fluconazole was also performed. Ononin showed better binding affinity than the antifungal agents (Table 1). Posaconazole is the native ligand of CaCYP51, and redocking of posaconazole was therefore performed to validate the docking protocol. The root mean square deviation (RMSD) between the predicted docking conformation and the observed X-ray crystal structure was 1.47 Å. Values less than 2 Å indicate that the docking protocol is valid. The binding free energies of all ligands are shown in Table 1.

**Figure 1:**
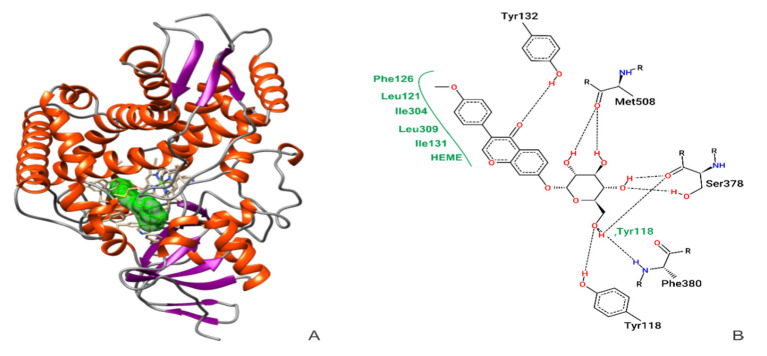
Molecular docking confirmation of ononin (in green) binding to CaCYP51 (PDB ID 5FSA) (**A**). Two-dimensional diagrams showing the connections of active amino acid residues of CaCYP51 with ononin. Black dashed line, hydrogen bonds; full green lines, van der Waals interactions (**B**).

Figure 1A shows the interaction between ononin and CaCYP51. Evaluation of the ononin + CaCYP51 complex obtained by MD showed that the ligand formed hydrogen bonds with residues Tyr132, Ser378, Phe380, and Met508, and hydrophobic connections with Tyr118, Leu121, Phe126, Leu131, Ile304, and Leu309, including interactions with the heme group (Figure 1B).

### 3.2. In Silico Analysis of the Biological Activities of Ononin and Its Prediction of Toxic Effects and Hepatotoxicity 

Table 2 shows the values for the probable activity (Pa) and probable inactivity (Pi) of ononin according to the Prediction of Activity Spectra for Substances (PASS) software. Several activities were predicted for Ononin, including anti-inflammatory, antioxidant, antibacterial, antimycobacterial, and hepatoprotective. The highest Pa value was obtained for the anti-infective activity (0942).

The prediction of pharmacokinetic characteristics by the SwissADME platform shows that ononin and fluconazole show high intestinal absorption, and they both were unable to across the blood–brain barrier (data not shown)

According to the predictions, ononin showed low toxicity for lethal doses (LD), the subcutaneous toxicity estimate was 6,079,000 mg/mL, the oral toxicity prediction estimate was 3,041,000 mg/kg, and it exhibited low intravenous and intraperitoneal toxicity; for this reason, this compound was classified as nontoxic (Class V (LD50 < 5000)) based on the Globally Harmonized Classification System (GHS) [20]. The predicted value of the intravenous toxicity of ononin was 1,406,000 mg/kg (Table 3).

The in silico predictions for hepatotoxicity are shown in Table 4. Ononin showed no toxicity to the analyzed cytochromes.

### 3.3. Minimum Inhibitory and Fungicidal Concentrations of Ononin

Table 5 shows the in vitro susceptibility of *Candida albicans* (ATCC 10231, CaS, and CaR). The MIC of ononin ranged from 3.9 to 7.8 µg/mL and the MFC from 15.6 to 62.5 µg/mL. The ATCC 10231 and CaS strains were susceptible to fluconazole, and the resistant clinical isolate (CaR) exhibited an MIC higher than 128 µg/mL, demonstrating its fluconazole resistance profile. The vehicle control (1% DMSO or less) had no effect on fungal growth.

### 3.4. Ononin Inhibited Candida albicans Growth

**Figure 2:**
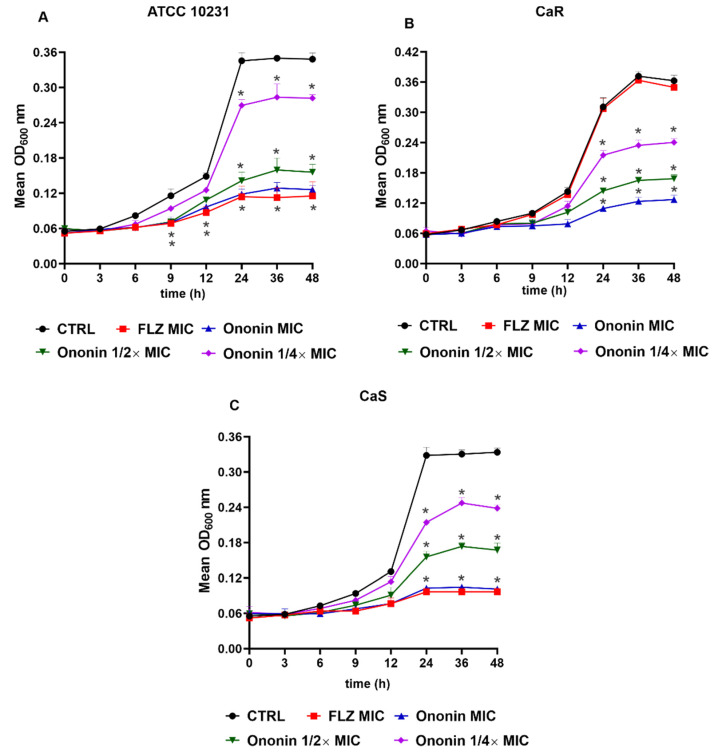
Ononin affected the growth kinetics of *Candida albicans*. Initial inoculum of 5 × 10^3^ CFU/mL. Growth curve of the ATCC 10231 reference strain (**A**). Fluconazole-sensitive isolate (CaS) (**B**). Fluconazole-resistant isolate (CaR) (**C**). Data are the mean ± standard deviation of three independent experiments. Statistical significance was determined by Student’s t-test. Significant at (*) *p* < 0.05 compared to the untreated control.

We evaluated the effect of ononin on the growth of *C. albicans* (ATCC 10231, CaS, and CaR) cultured for 48 h (Figure 2). Similar to fluconazole, ononin significantly inhibited the growth of *C. albicans* ATCC 10231 and CaS at the MIC after 6 h. In addition, a significant growth reduction was observed after 9 h for the two subinhibitory concentrations (Figure 2A,C). Analysis of the effect of ononin on the resistant clinical isolate (CaR) showed that the MIC of the compound was able to significantly reduce fungal growth after 6 h, 1/2 × MIC after 9 h, and 1/4 × CIM after 12 h compared to the control, and this effect persisted until the end of the experiment. As expected, fluconazole did not affect the resistant *C. albicans* isolate (CaR), which exhibited normal growth (Figure 2B). 

In the case of the two fluconazole-sensitive strains, the performance of ononin at its MIC was similar to that of the antifungal agent. The same profile was observed for the resistant isolate, demonstrating the efficacy of ononin against the strains tested (Figure 2). The fungal growth remained unchanged in cultures treated with DMSO (ononin diluent), and these results are similar to those of the negative untreated control (data not shown).

### 3.5. Ononin Inhibited the Development of Young and Mature Biofilms

**Figure 3:**
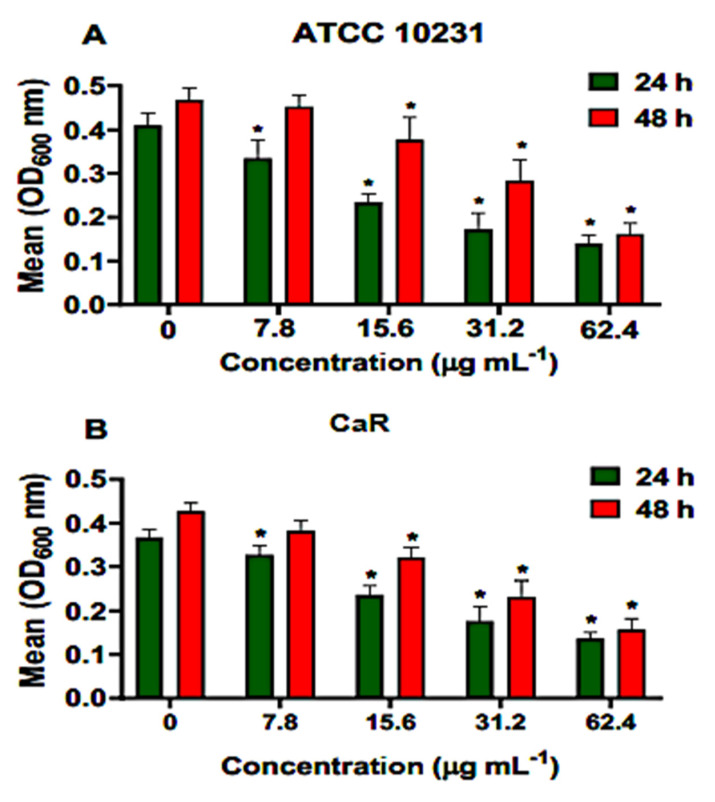
Ononin inhibited biofilm formation. Different concentrations of ononin (7.8, 15.6, 31.2, and 61.4 µg/mL) were added to the young biofilm (24 h) and to the mature biofilm (48 h), which was incubated with ononin for an additional 24 h. Mean OD600 values obtained for ATCC 10231 (**A**). Mean OD600 values obtained for fluconazole-resistant *Candida albicans* (CaR) (**B**). Data are the mean ± standard deviation. Statistical significance was determined by Student’s t-test (unpaired two-tailed, assuming unequal variance). (*) Statistically significant at *p* < 0.05 compared to the untreated control.

Different concentrations of ononin (7.8, 15.6, 31.2, and 61.4 µg/mL corresponding to the MIC, 2 × MIC, 4 × MIC, and 8 × MIC, respectively,) were used to treat adherent cell populations present in young and mature biofilms. Ononin significantly reduced the biofilm density of ATCC 10231 and CaR at the lowest dose (7.8 µg/mL), with a dose-dependent effect (Figure 3). The same dose-dependent effect of ononin was observed on mature biofilms. When the effect of ononin was evaluated by the colorimetric MTT assay using ATCC 10231, we observed that the metabolic activity of the young biofilm decreased by 72% and 81% at the concentrations of 4 × MIC and 8 × MIC, respectively, and that of the mature biofilm by 52% and 66% (Figure 3A). These data are consistent with the decrease in OD600 observed at the same concentrations (Figure 3).

**Figure 4:**
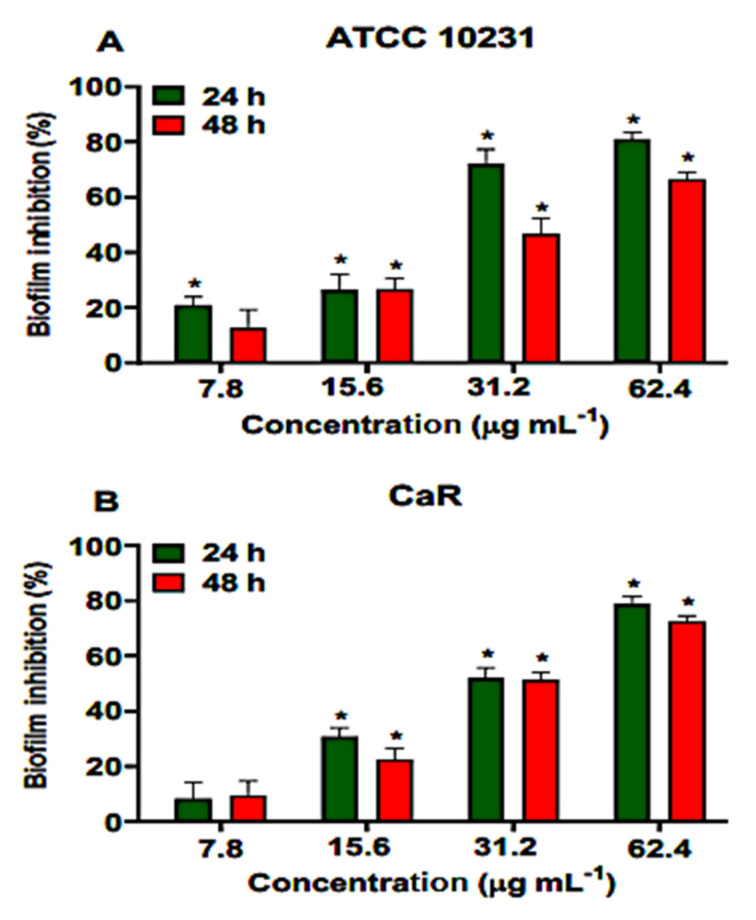
Effect of ononin inhibit the biofilm formation and metabolic activity. Different concentrations (7.8, 15.6, 31.2, and 61.4 µg/mL) of ononin were added to the young biofilm (24 h) and to the mature biofilm (48 h), which was incubated with ononin for an additional 24 h. ATCC 10231 reference strain (**A**). Fluconazole-resistant *Candida albicans* (CaR) (**B**). Biofilm formation and metabolic activity was determined by MTT. Data are the mean ± standard deviation. Statistical significance was determined by Student’s t-test (unpaired two-tailed, assuming unequal variance). (*) Statistically significant at *p* < 0.05 compared to the untreated control.

Ononin reduced more than 51% the metabolic activity of CaR young and mature biofilms with the concentration of 4 × MIC. At the highest concentration tested (62.4 µg/mL), this reduction was 78% in young and 72% in mature biofilms (Figure 4). DMSO (1%) showed similar results when compared to the untreated control (data not shown).

### 3.6. Ononin Shows Low Toxicity In Vitro and in Vivo

**Figure 5:**
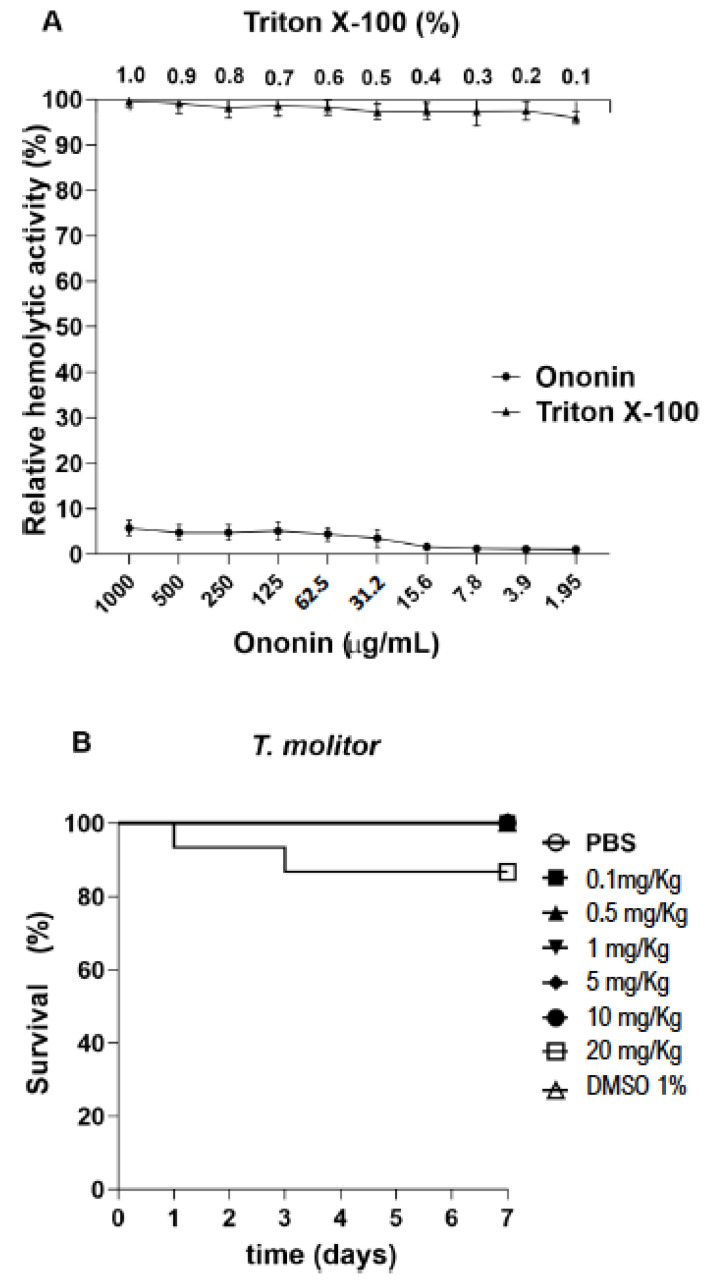
Ononin showed low toxicity in vitro and in vivo. Evaluation of cytotoxicity in sheep red blood cells exposed to ononin for 1 h. The cells were treated with different concentrations of Triton X-100 (0.1 to 1%) and ononin (1.95 to 1000 µg/mL) (**A**). *Tenebrio molitor* larvae were treated with a single ononin dose of 0.1 to 20 mg/kg (10 µL) (**B**). Controls were treated with 1% DMSO and PBS. The larvae were observed every 24 h for 7 days. Survival was 100% for concentrations up to 10 mg/kg and 86% for 20 mg/kg. Data are the mean ± standard deviation.

In vitro toxicity was evaluated using sheep red blood cells as the test system. The hemolytic potential of ononin was evaluated at different concentrations, none of which caused red blood cell lysis (Figure 5A). On the other hand, the positive control, Triton X-100, caused hemolysis at the tested concentrations. In addition, the injection of ononin in uninfected *T. molitor* larvae up to a concentration of 10 mg/kg did not alter the survival rate (Figure 5B).

### 3.7. Ononin Increases the Survival of Tenebrio molitor Infected with Candida albicans

**Figure 6:**
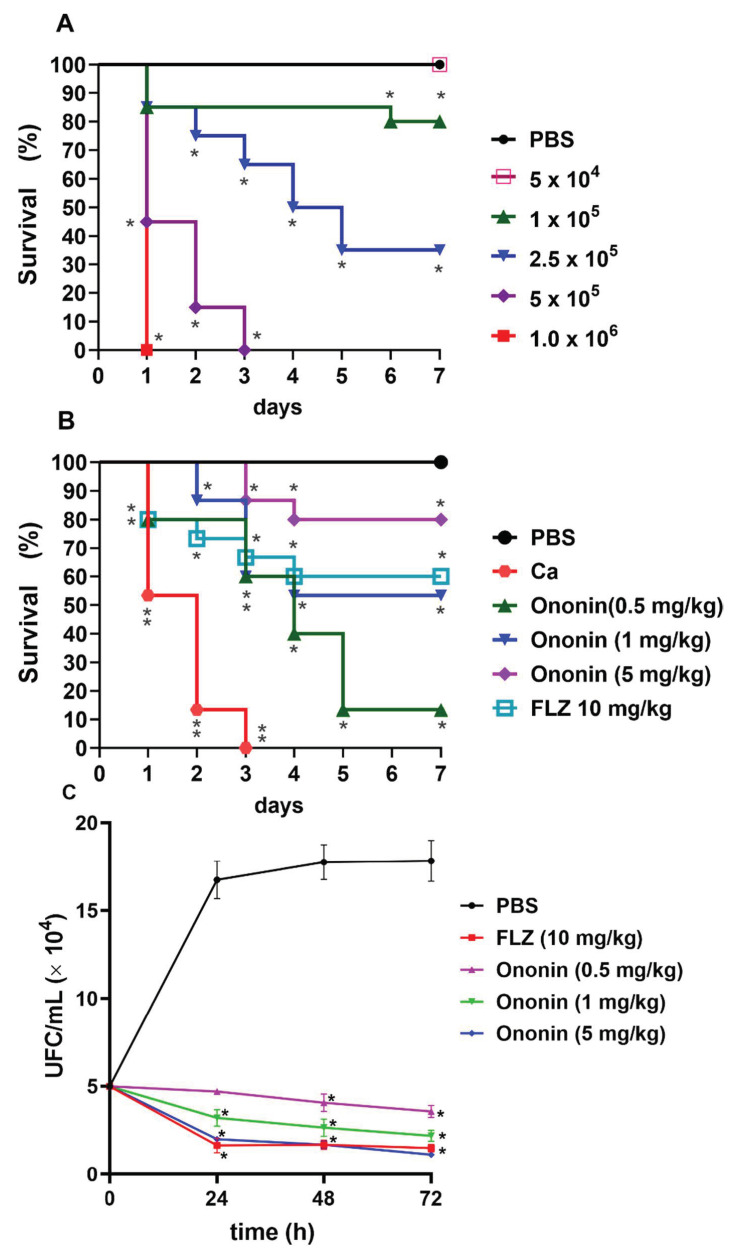
Ononin increased the lifespan in *Tenebrio molitor* infected with *Candida albicans*. *Tenebrio molitor* infected with different concentrations of *C. albicans* (ATCC 10231) for standardization of the inoculum used in the experiments (**A**). Ononin (0.5, 1, and 5 mg/kg) improves the survival of *T. molitor* during infection with a standardized *C. albicans* inoculum (5 × 10^5^ cells/larva) (**B**). Ononin reduces the number of *C. albicans* isolated from *T. molitor* (**C**). (*) *p* < 0.05 and (**) *p* < 0.01Indicates significant differences compared to infected and untreated larvae. FLZ, fluconazole.

We first investigated the most adequate concentration of *C. albicans* that was able to cause infection in the larvae. Our results show that *T. molitor* is susceptible to infection with *C. albicans* (Figure 6A). 

The mortality rate depended to the concentration of cells in the inoculum. The inoculum of 5 × 10^5^ cells/larva provided the most reproducible results, and was therefore used for the subsequent survival tests. In addition, the inoculum of 5 × 10^4^ cells/larva did not cause death, although infection persisted in the larvae throughout the period studied. To ensure that death was not due to shock caused by the injection of a large amount of fluid, one group of larvae was injected with 10 µL PBS as a control, and no deaths were observed (Figure 6A).

*Tenebrio molitor* infected with *C. albicans* (5 × 10^5^ cells/larva) exhibited 100% mortality on the third day, and the median survival of this group was 2 days (Figure 6B). The treatments affected the lifespan of the groups. Even at the lowest dose of ononin (0.5 mg/kg), the average survival was 4 days, with a survival rate of 13% after 7 days of infection. At the dose of 1 mg/kg, the survival rate was 53% on day 4 of infection, and the larvae started to die by day 2, with a delay in the onset of death compared to fluconazole (death started on day 1). At the dose of 5 mg/kg, the survival rate was 80% by day 4, with the onset of death on day 3. This result is superior to fluconazole, which ensured a survival rate of 60% of the larvae by day 4 at a dose of 10 mg/kg (Figure 6B). 

To determine whether mortality was related to the antifungal activity of ononin, the survival of *C. albicans* was evaluated in larvae using a previously standardized nonlethal inoculum (Figure 6C). We observed elevated fungal loads in untreated infected larvae on the days of the tests. When treated with ononin at all concentrations, the animals exhibited significantly lower CFU/mL counts on all days evaluated (*p* < 0.05) when compared to the untreated control. There was no significant difference between 10 mg/kg fluconazole and 5 mg/kg ononin. All animals of the control groups treated with PBS or treated only with ononin up to the highest concentration (10 mg/kg) survived, demonstrating that larval death in the experiment was due to infection.

## 4. Discussion

*Candida albicans* species can cause superficial infection of the oral and vaginal mucosa, as well as infections disseminated to the bloodstream and deep tissues. The commonly prescribed antifungal agent for most *C. albicans* infections is fluconazole, a member of the azole class [1,40]. Its pharmacological effect is related to the inhibition of the cytochrome P450 enzyme lanosterol 14α-demethylase (CYP51), which is encoded by the ERG11 gene [41]. Azoles directly inhibit the biosynthesis of ergosterol by binding to *C. albicans* CYP51 (CaCYP51), causing the depletion of ergosterol and, at the same time, increasing 14α-methylated sterols, which leads to the accumulation of toxic sterol intermediates [1,42]. 

Many plants are used in research to identify new bases of antifungal agents [43]. The results of biological tests conducted by our group show that the hydroethanolic extract and ethyl acetate fraction of *Platonia insignis* Mart. possesses antifungal activity against several *Candida* species, including 16 clinical isolates. Both the extract and its fractions showed antifungal effects against *Candida* isolates, with MICs ranging from 1.8 to 6.3 mg/mL against *C. albicans* and 1.6 to 8.3 mg/mL against *C. glabrata*. [10]. In the present study, the main compounds identified in the extract (quinic acid, ononin, orientin, vitexin, and fukugentin) were submitted to MD to evaluate their possible interaction with CaCYP51. Negative binding free energies indicate that these interactions favor formation of the ligand–receptor complex, and the inhibitory constant is a valuable piece of data to predict clinically relevant drug interactions [28]. According to the MD results, among the compounds identified in the extract, ononin exhibited the most favorable parameters for the formation of complexes with CaCYP51, and was therefore selected for this study.

The X-ray crystallography data of the CaCYP51–itraconazole complex (PDB ID 5V5Z) obtained in previous studies showed that this drug interacts with amino acid residues Gly307, Thr311, and Leu376. Our results indicate that ononin interacts with the same amino acids, as well as with the neighboring residues Ile304, Leu309, Ser378, and Phe380 (Figure 1). Previous results observed for the drug VT-1161 (oteseconazole) (PDB ID 5TZ1) complexed with CaCYP51, showed interactions with the same residues as those found in our study. This new class of antifungals targeting fungal CYP51 without significantly affecting the human homolog is promising to increase treatment options [44]. It is important to mention that posaconazole, used as control in our study, interacts with Leu121, Phe126, Ile131, Phe228, and Leu376, the same residues identified for ononin, suggesting that the latter effectively binds to the active site of CaCYP51 (Table 1).

Molecular docking studies using fluconazole have demonstrated the unsatisfactory affinity of this drug to CaCYP51 when compared to other conventional antifungal agents [6]. In a study testing fluconazole and posaconazole in vitro against *C. albicans*, fluconazole exhibited low efficiency (activity of 54%), while posaconazole showed very high efficiency of 98% when tested at the same concentration [45]. These results agree with our in silico data, demonstrating a superior binding affinity for posaconazole compared to fluconazole (Table 1).

Ononin and fluconazole showed high intestinal absorption characteristics, but they both are not able to cross the blood–brain barrier according the SwissADME in silico prediction (Table 3). In addition, the in silico analysis revealed that ononin has no mutagenic, tumorigenic, irritating, or reproductive system effects (Table 3, and Table 4). In silico prediction of ononin include anti-inflammatory; antioxidant, anti-infective, antifungal, and immunostimulant biological proprieties (Table 2). The low toxicity of ononin was also confirmed by our in vivo results using *T. molitor*, since all animals remained alive during the period of observation even when they received the highest dose of this compound (20 mg/Kg, Figure 5)

The anti-inflammatory effect of ononin was demonstrated in vitro on lipopolysaccharide-induced inflammatory responses using RAW 264.7 [15] and in vivo in rats with Alzheimer’s disease (AD), a chronic neurodegenerative disease categorized by the deficiency in the cognition and memory. According to Chen et al. [46], ononin treatment effectively modulates behavioral alterations, ameliorates the cognitive impairment, and suppresses the neuroinflammation oxidative stress in AD animals. Ononin also showed an anti-inflammatory effect in rheumatoid arthritis. In this case, the compound induced cell apoptosis and reduced the fibroblast-like synoviocyte inflammation by alleviating MAPK and the NF-κB signaling pathway [47]. In relation to the anti-infective and antifungal effects of ononin, there is limited information; for this reason, it was our aim to investigate this activity in vitro and in vivo. 

The present study evaluated the in vitro antifungal activity of ononin against two clinical *C. albicans* isolates obtained from patients with vulvovaginal candidiasis (one sensitive, CaS, and one resistant to fluconazole, CaR) and with ATCC 10231 as a reference strain. Ononin inhibited the growth of *C. albicans* (ATCC 10231, CaR, and CaS) with MICs of 3.9 to 7.8 µg/mL, and was effective for the both strains: fluconazole-sensitive and -resistant isolates (Table 5). For the CaR isolate, there was no difference in growth between the untreated control and the cultures treated with fluconazole, confirming the resistant genotype. Instead, analysis of growth kinetics showed a dose-dependent effect for the compound, which also exhibited activity even when used at subinhibitory concentrations (Figure 2). 

The emergence of *C. albicans* strains that are resistant to azoles as a result of the prophylactic and prolonged use of azole antifungal agents in clinical practice, especially fluconazole, has been reported [48,49]. This has become a growing problem, especially among immunocompromised individuals, such as transplant patients, patients with cancer, and HIV/AIDS patients [50]; for this reason, there is an urgent need to develop new effective therapeutic strategies against these strains that are resistant to conventional antifungals.

In view of the positive results obtained in silico and the effect of ononin on planktonic *C. albicans* cells, we studied the effect of this substance on young and mature biofilms. The results show that ononin was effective against the two types of biofilms, since ononin reduced the density of young biofilms at the lowest concentration tested, as well as the density of mature biofilms at a concentration of 2 × MIC or higher (Figure 3). These data were validated by the MTT assay, which showed considerable inhibition of young and mature biofilms (*p* < 0.05), with the reduction of metabolic activity reaching 52 to 81% (Figure 4). Thus, ononin is a potential antifungal candidate since it inhibited biofilm formation, as well as the mature biofilm, which is normally difficult to treat.

Biofilm formation is an important virulence factor of *C. albicans* that contributes to the pathogenicity of this species. Biofilms consist of a structured set of cells, and can develop at different locations in the human body [37]. Antifungal therapy is necessary for successful treatment and control of the infection [51]. An important characteristic of *Candida* biofilms is their resistance to antifungal agents, which can be classified as intrinsic or acquired through the transfer of genetic material between biofilm cells [52]. In addition, these biofilms resist the host immune response [2], a fact that contributes to the persistence of yeasts in tissues and on implanted medical devices and to therapeutic failures. 

The capacity to form biofilms is a marked and clinically relevant characteristic of *C. albicans* virulence [53]. Our results show a more effective activity of ononin against young biofilms; moreover, its effect on mature biofilms was also significant, suggesting the possibility to consider the ononin as an alternative to conventional antifungal agents and a target in the development of new drugs. Biological properties of ononin, such as antioxidant, anti-inflammatory, antiviral, and antibacterial activity, have already been reported [14,15,18,20]; however, this is the first study to analyze its antifungal activity against *C. albicans*.

Molecular docking studies have proved efficient for the identification of new antifungal drug candidates. In vitro studies using 3,4-dichlorobenzyl derivatives against *C. albicans* demonstrated that the derivative with the best results exhibited comparable or superior activity to the conventional drugs tested, including fluconazole, and the same compounds provided the best binding affinity in MD, corroborating with our in vitro data [54,55].

In view of these findings, we evaluated the efficacy of ononin in vivo using the *T. molitor* as a model of infection with *C. albicans*. The use of invertebrate for the study of microbial infection has increased considerably in an attempt to avoid or reduce the use of vertebrates in animal experimentation [38,39,56].

*Tenebrio molitor* (Coleoptera), popularly known as the mealworm, is found worldwide, and is a rapid, inexpensive, and efficient model for in vivo testing of antifungal substances, since it is susceptible to human pathogens such as *C. albicans*, can be incubated at 37 °C, and its size favors the collection of body fluids [56]. Within this context and considering the in silico and in vitro results, we first standardized the most appropriate inoculum for the subsequent experiments. Our data agree with the inocula used in *T. molitor* experimentation [38,57,58]. Ononin extended the lifespan of the larvae and reduced fungal loads (Figure 6). The lowest concentration of the compound was able to increase larval survival and ensured an average survival of 3 days, while the median survival of the untreated infected control was only 2 days. Survival at the higher ononin concentration used for treatment (5 mg/kg) was superior to that observed for fluconazole. It should be noted that ononin did not exert any toxic effect on *T. molitor* survival when using concentrations higher than 10 mg/kg (Figure 5). The absence of hemolytic activity of ononin at higher concentrations corroborates these data, thus demonstrating that ononin can be used safely in vivo since it is not toxic. Mammalian red blood cells represent a good model for assessing the cytotoxicity of organic and inorganic natural or synthetic molecules by measuring cell damage [59].

Taken together, the present findings show the high affinity of ononin for CaCYP51 in silico, its effective activity against biofilms, and the reduction of fungal loads in treated animals, in addition to the absence of toxicity, demonstrating the significant therapeutic potential of this compound.

## 5. Conclusions

This study demonstrates the antifungal activity of ononin against *C. albicans* species with different genotypes, since the compound was able to reduce the density and viability of biofilms, which might be related to a reduction in the activity of cells inside the biofilm. Ononin was not cytotoxic, and also showed several biological activities that may improve the antifungal effect in vivo and can be associated with the efficacy in increasing the *T. molitor* survival. This finding suggests that the mechanism of action of ononin might be related to the biosynthesis of ergosterol, rendering it a strong antifungal candidate.

## Figures and Tables

**Table 1:** Binding free energies and inhibition constants docking between the compounds identified in the extract of *Platonia insignis* to CaCYP51.

Ligand	ΔGbind (kcal/mol) *	Ki (μM) **
Ononin	−10.89	0.01
Orientin	−6.48	17.79
Vitexin	−5.75	61.49
Quinic acid	−4.39	602.41
Fukugentin	−1.32	1080.40
Posaconazole	−8.75	0.38
Fluconazole	−6.14	31.61

* ΔGbind, binding free energy; ** Ki, inhibition constant calculated in silico.

**Table 2:** In silico analysis of the biological activities of ononin.

Activity	Pa ^a^	Pi ^b^
Anti-infective	0.942	0.003
Hepatoprotectant	0.913	0.002
Antioxidant	0.711	0.004
Antifungal	0.648	0.014
Anti-inflammatory	0.64	0.024

(a) Pa, probable activity; (b) Pi, probable inactivity.

**Table 3:** Predicted acute toxic effects of ononin in silico.

Parameter	Ononin
Oral Toxicity (LD50 mg/Kg)	3,041,000
Intravenous Toxicity (LD50 mg/Kg)	1,406,000
Subcutaneous Toxicity (LD50 mg/Kg)	6,079,000
Intraperitoneal Toxicity (LD50 mg/Kg)	652,600

**Table 4:** In silico prediction of chemical toxicity and inhibitory effect for the hepatic cytochromes.

Cytochrome	Ononin	Toxicity
CYP1A2	0.994 ^a^	NT
CYP2C19	0.982 ^a^	NT
CYP2C9	0.879 ^a^	NT
CYP2D6	0.950 _a_	NT
CYP3A4	0.984 ^a^	NT

NT, nontoxic; T, toxic; (a) 0.7–0.9, no expected toxicity.

**Table 5:** Minimum inhibitory concentration (MIC) and minimum fungicidal concentration (MFC) of ononin against *Candida albicans*.

C. Albicans Strains	Ononin		Fluconazole	
	MIC (µg/mL)	MFC (µg/mL)	MIC (µg/mL)	MFC (µg/mL)
ATCC 10231	7.8	31.2	0.5	16
CaS	3.9	15.6	0.25	8
CaR	7.8	62.5	>128	>128

ATCC, American Type Culture Collection; CaS, fluconazole-sensitive *C. albicans*; CaR, fluconazole-resistant *C. albicans.*

## Data Availability

Not applicable.

## Supplementary Materials

The following supporting information can be downloaded at: https://www.mdpi.com/article/10.3390/metabo12111014/s1, Figure S1: Structure of ononin; Table S1: The physicochemical properties of ononin and fluconazole.
