# Supplementary material for: Anti-Candida albicans Activity of Ononin and Other Secondary Metabolites from Platonia Insignis MART
Source: Metabolites. 2022 Oct 24;12(11):1014. doi: 10.3390/metabo12111014 (PMC9696916; doi:10.3390/metabo12111014)
Supplement: Supplementary file 1 [file metabolites-12-01014-s001.zip › metabolites-1952118-supplementary.pdf]

Supplementar material

The structure of ononin is shown in Figure S1 and the physicochemical properties of ononin and fluconazole are shown in Table S1.

Figure S1. Structure of ononin.

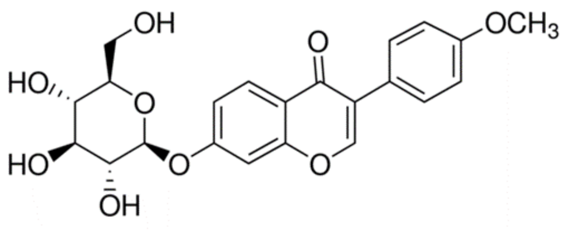

Table S1. The Physicochemical Properties of Ononin and Fluconazole:

Properties	Ononin	Fluconazole
Formula	$C_{22}H_{22}O_9$	$C_{13}H_{12}F_2N_6O$
Molecular weight (g/mol)	430.40	306.27
Nº heavy atoms:	31	22
Nº aromatic heavy atoms	16	16
Fraction Csp3:	0.32	0.23
Nº rotatable bonds	5	5
Nº H-bond acceptors	9	7
Nº H-bond donors	4	1
Molar Refractivity	108.56	70.71
Topological Polar Surface área (Å²)	138.82	81.65
